# CDPNet: a deformable ProtoPNet for interpretable wheat leaf disease identification

**DOI:** 10.3389/fpls.2025.1676798

**Published:** 2025-11-06

**Authors:** Jinyu Zeng, Bingjing Jia, Chenguang Song, Hua Ge, Lei Shi, Bo Kang

**Affiliations:** 1 College of Information and Network Engineering, Anhui Science and Technology University, Bengbu, Anhui, China; 2 State Key Laboratory of Media Convergence and Communication, Communication University of China, Beijing, China

**Keywords:** identification of wheat leaf diseases, interpretability, CDPNet, Cross Attention, Barlow Twins

## Abstract

**Introduction:**

Accurate identification of wheat leaf diseases is crucial for food security, but existing prototype-based computer vision models struggle with the scattered nature of lesions in field conditions and lack interpretability.

**Methods:**

To address this, we propose the Contrastive Deformable Prototypical part Network (CDPNet). The idea of CDPNet is to identify key image regions that influence model decisions by computing similarity measures between convolutional feature maps and latent prototype feature representations. Moreover, to effectively separate the disease target area from its complex background noise and enhance the discriminability of disease features, CDPNet introduces the Cross Attention (CA) Mechanism. Additionally, to address the scarcity of wheat leaf disease image data, we employ the Barlow Twins self-supervised contrastive learning method to capture feature differences across samples. This approach enhances the model's sensitivity to inter-class distinctions and intra-class consistency, thereby improving its ability to differentiate between various diseases.

**Results:**

Experimental results demonstrate that the proposed CDPNet achieves an average recognition accuracy of 95.83% on the wheat leaf disease dataset, exceeding the baseline model by 2.35%.

**Discussion:**

Compared to other models, this approach delivers superior performance and provides clinically interpretable decision support for the identification of real-world wheat diseases in field settings.

## 1 Introduction

Wheat is one of the three major global food crops, ranking among the highest in both production volume and cultivated area. Its widespread cultivation and stable yields serve as a critical safeguard for global food security. However, disease infestation throughout its growth stages remains the primary challenge limiting stable and high yields (Bao et al., 2021a). Statistics show that leaf diseases, such as leaf blight, mildew, and rust, can lead to annual global wheat yield losses ranging from 10% to 30%. These diseases not only lead to direct yield reductions but also trigger secondary hazards, such as grain quality deterioration and mycotoxin contamination, causing substantial losses in agricultural production (Simón et al., 2021). Therefore, accurate identification of wheat diseases, particularly leaf diseases, is critical for implementing effective control measures and ensuring healthy growth to enhance yields (Nigam et al., 2023).

With advancements in modern technology, machine learning and deep learning techniques are increasingly being applied to crop pest and disease detection. These techniques have shown highly promising results in achieving precise identification of crop pests and diseases using computer vision technology (Deng et al., 2025). Traditional machine learning techniques, such as Support Vector Machines (SVM) (Rezvani and Wu, 2023), Random Forests (Gao et al., 2022), and Decision Trees (Alaniz et al., 2021), have been widely employed in wheat disease detection. These techniques employ various algorithms to extract different features from images, including color, texture, and shape (Syazwani et al., 2022). The extracted features are subsequently used to train an image classifier capable of accurately distinguishing between healthy and diseased wheat. (Khan et al., 2023a) developed an automatic classification framework for wheat diseases based on machine learning techniques, effectively identifying wheat brown rust and yellow rust. (Bao et al., 2021b) presented an approach for detecting leaf diseases and their severity based on E-MMC metric learning, focusing on wheat mildew and stripe rust. However, in machine learning-based algorithms for identifying crop leaf pests and diseases, traditional image processing techniques or manually designed feature-based classification and recognition algorithms are commonly employed (Zhang et al., 2023). These algorithms are typically limited to extracting low-level features and struggle to capture deep and complex image information, failing to fully capture the complexity of sample data, which affects the accuracy of diagnosing localized regions of leaf diseases (Xu et al., 2024).

Recently, deep learning has made significant advancements in the field of crop pest and disease identification, achieving remarkable success in domains such as image processing (Chen et al., 2021b), natural language processing (Zeng and Xiong, 2022), and speech recognition (Kim et al., 2024), owing to its powerful representational capabilities (Zhang et al., 2024; Khan et al., 2023b). Advanced deep learning techniques, such as convolutional neural networks (CNNs) and attention mechanisms, have been applied to crop pest and disease detection (Jia et al., 2023). These methods can automatically, efficiently, and accurately extract target features from large datasets of crop leaf pest and disease images, thereby replacing traditional recognition approaches that rely on manual feature extraction. To facilitate rapid and accurate identification of wheat leaf diseases and reduce agricultural losses, (Jiang et al., 2021) introduced an enhanced VGG16 model integrated with a multi-task transfer learning strategy for detecting wheat leaf diseases. They modified the VGG16 model and employed a pre-trained model on the ImageNET platform for transfer learning and interactive learning. Experimental results demonstrated that this method outperformed single-task models, the ResNet50 model, and the DenseNet121 model. (Dong et al., 2024) presented the SC-ConvNeXt model for wheat disease identification. This network model utilizes ConvNeXt-T for feature extraction and incorporates an enhanced CBAM mechanism to mitigate the effects of interference from complex environmental factors. To improve the accuracy of a single category of wheat disease identification, (Nigam et al., 2023) focused solely on wheat rust and fine-tuned the EfficientNet B4 model for wheat disease recognition. (Chang et al., 2024) proposed the Imp-DenseNet model for identifying the three types of wheat rust, aiming to facilitate wheat rust identification in field environments. (Hassan et al., 2024) advanced the UNET detection model for yellow rust disease detection in wheat, achieving high classification accuracy for wheat diseases.

Deep learning constructs multi-layer neural network models that enable advanced data representation and understanding through hierarchical feature extraction and abstraction. However, as these multi-layer networks become deeper, each layer introduces numerous parameters and nonlinear activation functions (Chang, 2025). Although such architectures excel in handling complex data and tasks, their high complexity and nonlinearity lead to low transparency and poor interpretability (Goethals et al., 2022). Users often struggle to intuitively understand the logical basis behind model decisions, casting doubt on their credibility and perceiving deep models as data-driven “black box” systems (Marcus and Teuwen, 2024). The decision-making process in such models inherently involves high-dimensional nonlinear mappings, with internal reasoning mechanisms that lack explicit interpretability. This fundamentally complicates result attribution and causal inference. In agricultural applications, such as leaf disease identification, researchers have proposed various interpretability methods. These techniques such as feature visualization, attention mechanism analysis, and decision rule extraction (Hernández et al., 2024) aim to unveil the internal reasoning pathways of deep models during disease diagnosis, thereby enhancing model transparency and credibility.

However, most existing intrinsically interpretable models rely on spatially rigid prototypes, which are unable to explicitly explain the geometric changes in disease patterns and complex background feature information. This limitation restricts the provision of detailed explanations and improved recognition accuracy (Ma et al., 2024). Therefore, in this work, we propose an interpretable wheat leaf disease identification model (CDPNet) based on a deformable prototypical part network and contrastive learning. In CDPNet, each prototype comprises multiple prototypical parts that adaptively adjust their spatial positions relative to one another depending on the input image. This allows each prototype to detect object features with greater tolerance of spatial transformations, since the parts within a prototype can move. To identify wheat leaf disease types and uncover the infected regions influencing model decisions, we first employ Deformable ProtoPNet (Donnelly et al., 2022) to calculate the similarity values relating to the convolutional feature maps of the image and the latent prototype features. Generally, a higher similarity score indicates a greater influence of that region on the model’s decision. Secondly, to effectively distinguish the target regions of wheat leaf diseases from complex backgrounds and enhance the model’s feature extraction capabilities, we introduce the CA Mechanism (Lin et al., 2022; Chen et al., 2021a). This mechanism guides the model to focus on spatial contextual features. By amplifying differences between disease areas and surrounding backgrounds, it significantly enhances the discriminative power of disease features, thereby improving recognition performance in complex scenarios. Finally, in practical applications, some wheat leaf diseases exhibit low incidence rates and high image acquisition costs, leading to limited training data. To address this challenge, we introduce the self-supervised contrastive learning strategy Barlow Twins (Zbontar et al., 2021). This approach maximizes similarity between different transformed versions of the same image while minimizing similarity between distinct images, thereby enabling deep exploration of discriminative features across wheat leaf disease instances. In summary, the main contributions of this work are summarized as follows:

- The deformable prototype network in CDPNet is designed to adaptively adjust relative spatial positions through flexible and dynamic prototype learning, thereby providing clinical interpretability for the identification of wheat leaf diseases.
- We propose a novel interpretable model for wheat leaf disease identification—the Contrastive Deformable Prototypical part Network (CDPNet). This model is capable of discovering key regions in wheat leaf disease images that influence the model’s decisions. Additionally, it effectively distinguishes between disease target regions and complex backgrounds, and deeply mines latent feature information among samples, offering a more comprehensive and in-depth analytical perspective for disease identification.
- We have created a real-world wheat leaf disease dataset to facilitate further research on disease identification in practical field environments.
- Through extensive experimentation using the wheat leaf disease dataset, as well as other public crop disease datasets, the results demonstrate that CDPNet achieves superior identification performance, outperforming classical models, and validating its generalization ability and interpretability.

## 2 Related work

### 2.1 Leaf disease identification based on machine learning

The recognition of crop leaf diseases has long been a central research focus within the field of agricultural engineering (Thakur et al., 2022). The application of modern information technologies for diagnosing and identifying crop leaf diseases provides an advanced, systematic, and effective approach (Balakrishna and Rao, 2019). Research on leaf disease identification methods can be broadly categorized into two primary approaches: traditional machine learning techniques and contemporary deep learning approaches.

Machine learning is utilized to automatically analyze large-scale datasets, uncover latent patterns, and apply these insights to subsequent analysis and prediction tasks. With the advancement of image processing technologies, machine learning has been extensively applied to leaf disease identification (Thakur et al., 2022). Researchers employ feature extraction and segmentation techniques to capture key disease characteristics, which are subsequently classified using machine learning algorithms. Under conditions of limited computational resources, machine learning initially emerged as the primary research tool, producing notable results. (Balakrishna and Rao (2019) conducted experiments on tomato leaf diseases, initially categorizing tomato leaves into healthy and diseased classes using the K-Nearest Neighbors (KNN) method, followed by effective sub-classification of diseased leaves using a combination of Probabilistic Neural Networks (PNN) and KNN. (Pattnaik and Parvathi, 2021) utilized the Histogram of Oriented Gradients (HOG) to characterize features extracted from segmented images, which were then input into a Support Vector Machine (SVM) for classification. Due to the relatively low classification difficulty, their test accuracy reached 97%. (Javidan et al., 2023) utilized K-means clustering technology to locate infected regions in images and subsequently accomplished grape leaf disease classification through SVM. However, machine learning-based approaches to leaf disease recognition, while capable of distinguishing certain disease features and generating classification results, continue to exhibit several limitations (Wani et al., 2022): (1) Feature selection limitations: Traditional machine learning approaches require the manual selection of features to describe pest or disease images. However, such features often capture only partial image information. Moreover, the variability of pests and diseases across growing environments renders selected features insufficient to comprehensively represent all relevant characteristics. (2) Feature extraction challenges: Machine learning cannot automatically extract features, necessitating manual extraction, which is also highly sensitive to image noise. (3) Limited generalizability and recognition scope: Trained models can typically recognize only the specific crop pests and diseases on which they were trained, making it difficult to extend recognition capabilities to other disease types. (4) Narrow application scope: Constrained by disease-specific characteristics, these methods are generally limited to learning and classifying features of particular crops in specific regions, which restricts their applicability across a broader range of species.

Compared with traditional machine learning methods, deep learning addresses inefficiencies and low accuracy arising from manually designed features in complex environments. In recent years, alongside the ascent of deep learning advancements, CNNs and Transformers have undergone rapid development (Khan et al., 2023a). The convolutional layers of CNNs utilize a local receptive field design, in which each neuron is connected only to a restricted region of the input image (Quan et al., 2022). This design is well-suited to image data, since local information (e.g., edges, textures) plays a critical role in object recognition (Xu et al., 2024). (Bao et al., 2022) presented an enhanced recognition network called AX-RetinaNet. This model employs an X-module enhanced multi-scale feature integration and channel attention for feature extraction, thereby enabling effective detection and classification of tea diseases, with an identification accuracy reaching 96.75%. To address the issue of abnormal recognition caused by various image distortions in the healthy and diseased parts of coffee plant leaves, (Nawaz et al., 2024) suggested a CoffeeNet model. The model under consideration makes use of a ResNet-50 framework and an attention mechanism for the purpose of extracting features of diverse coffee leaf diseases. To increase the accuracy of classifying plant leaf diseases while keeping the model lightweight, (Zhao et al., 2024) developed a neural architecture termed CAST-Net. This lightweight network model is based on a combination of convolution and self-attention. It further employs a self-distillation method to enhance the precision of leaf disease classification while reducing model parameters and failure cases. The findings indicate that, in comparison with existing models, CAST-Net attains enhanced precision, reduced parameter complexity, decreased training time, and lower computational complexity. The Transformer architecture captures global dependencies among elements of input sequences (Khan et al., 2022b). In image classification, the self-attention mechanism allows the model to incorporate information from all pixels or features when processing each individual one (Xu et al., 2021). This enables Transformers to more effectively capture the overall structure and contextual information of images, providing advantages for classification tasks that rely on global information. (Borhani et al., 2022) proposed a lightweight model based on Vision Transformer for plant disease classification. To better 174 leverage the strengths of both CNNs and Transformers, (Alshammari et al., 2022) utilized a deep ensemble learning strategy to combine a CNN with a vision transformer model for the purpose of classifying Olive Diseases. (Thakur et al., 2023) also proposed a composite model that integrates the advantages of ViT with the innate feature extraction capabilities of CNNS for plant leaf disease recognition.

### 2.2 Interpretable leaf disease classification using deep neural networks

Image classification, a fundamental task in computer vision, focuses on achieving accurate multi-class categorization based on image content while minimizing error. Machine learning initially demonstrated significant potential in image classification, and within this domain, deep learning gradually emerged as the more suitable approach. CNNs, characterized by local connectivity and translation invariance, align well with the inherent properties of image data. Despite continual improvements in classification accuracy, researchers have identified persistent challenges in deep learning for image tasks, including adversarial robustness, generalization, and fairness. Interpretability research provides a critical pathway to address the “black box” nature of deep learning (Zhang et al., 2025). Its objective is to elucidate model decision-making mechanisms through human-understandable methods, thereby enhancing credibility and robustness. From a modeling perspective, Interpretability research can be broadly categorized into two types: *post-hoc* interpretation methods and intrinsically interpretable models.

1. *Post-hoc* interpretation methods. These primarily target black-box models, analyzing them through various algorithms such as visualization analysis, importance analysis, etc., to infer the model’s decision-making procedure. Examples include Feature Attribution, Permutation Importance, and Class Activation Mapping (CAM). For instance, (Mishra et al., 2024) proposed an image-based interpretable leaf disease detection framework (I-LDD) that utilizes Local Interpretable Model-agnostic Explanations (LIME) to obtain explanations for model classifications. Similarly, (Raval and Chaki, 2024) employed LIME technology, taking leaf diseases as an example, and (Chakrabarty et al., 2024) used interpretable artificial intelligence to visualize the decision-making processes of their model, focusing on rice leaf diseases. To offer a more thorough understanding of the model’s interpretability, (Hernández et al., 2024) adopted the Grad-CAM method to visualize the infected regions of grape leaves, explaining the neural network’s attribution to leaf disease detection. (Wei et al., 2022) presented the ResNet-CBAM model for interpretable leaf disease classification and compared three visualization methods: SmoothGrad, LIME, and GradCAM, to conduct *post-hoc* interpretability of the model. Meanwhile, (Dai et al., 2024) employed t-SNE and SHAP visualization methods to explain whether the model focuses on plant pest and disease characteristics.
2. Intrinsically interpretable models. Intrinsically interpretable models require us to select human-understandable features and adopt models with good interpretability during the problem-solving process (Jiang et al., 2025). This objective is realized through the construction of models that are self-explanatory and which incorporate interpretability directly into their structures. Such models include decision trees, rule-based models, linear models, and attention models. Our model belongs to the category of intrinsically interpretable models, which integrate interpretability into the specific model structure, enabling the model itself to possess interpretability. The model outputs not only the results but also the reasons behind those results, thereby ensuring the reliability and safety of the interpretations. CDPNet discovers key regions influencing model decisions and predicts pest and disease categories by computing similarity of the convolutional feature maps of images to the latent prototype features, thus explaining the model’s decision-making process and attribution. Through flexible and dynamic prototype learning, it achieves accurate identification of wheat leaf diseases in natural field environments along with rich interpretability.

## 3 Materials and methods

### 3.1 Dataset acquisition and image preprocessing

**Figure 1:**
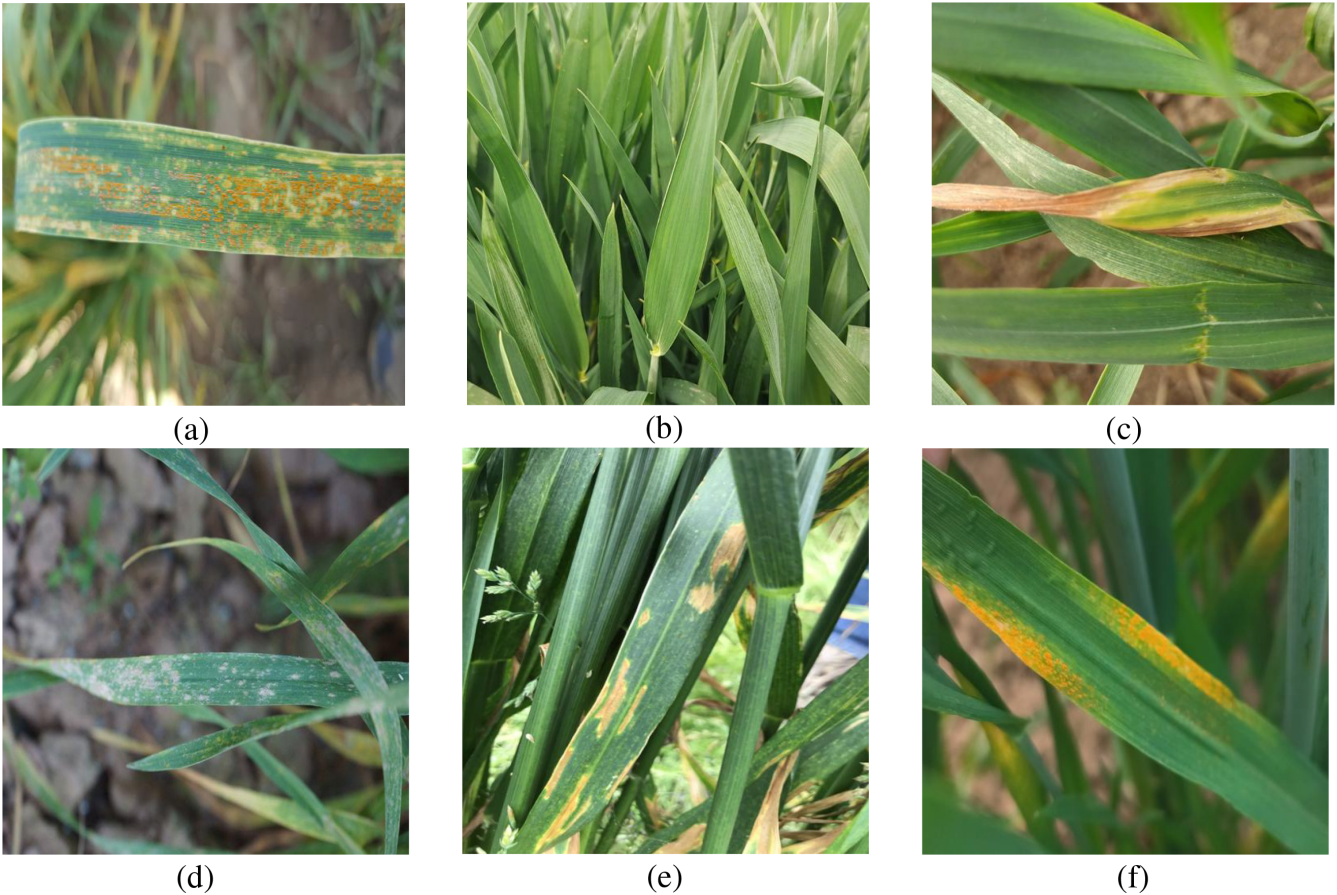
Samples of wheat leaf disease. **(a)** Brown Rust, **(b)** Healthy, **(c)** Leaf Blight, **(d)** Mildew, **(e)** Septoria, **(f)** Yellow Rust.

This study utilized a hybrid data source to construct a wheat leaf disease dataset. The self-constructed dataset was compiled by the research team under expert guidance through field photography conducted in Fengyang County, Chuzhou City, Anhui Province, from April 15 to May 15, 2024. Fieldwork was conducted daily between 8:00 AM and 6:00 PM. Images were captured using a Vivo Y70s smartphone, covering six common wheat leaf diseases: Brown Rust, Healthy, Leaf Blight, Mildew, Septoria, and Yellow Rust. A total of 1,340 valid images were obtained. **Figure 1** illustrates images of wheat leaf diseases from various categories.

To enhance the dataset, this study also incorporated wheat leaf disease images from the Wheat Plant Diseases dataset on Kaggle. This dataset is designed to enable researchers and developers to build robust machine learning models for classifying various wheat plant diseases. It provides a collection of high resolution images depicting real-world wheat diseases without relying on artificial augmentation techniques. Data filtering was performed on this dataset to remove duplicate and misclassified images from the original public dataset. This process resulted in the creation of a wheat leaf disease dataset (WL-Disease) comprising six categories and a total of 6,513 images. The specific categories and their corresponding image counts are detailed in **Table 1**.

**Table 1:** Detailed descriptions of the various types of samples within the WL-disease dataset.

Category	Number	Train set	Test set
Brown Rust	1054	843	211
Healthy	812	645	167
Leaf Blight	1008	806	202
Mildew	1328	1062	266
Septoria	916	732	184
Yellow Rust	1395	1116	279
Total number	6513	5204	1309

In the WL-Disease dataset, all training images are labeled without annotations on specific image regions. The dataset was randomly divided into training and testing sets at an 80:20 ratio to ensure the validity and fairness of model training and validation.

**Figure 2:**
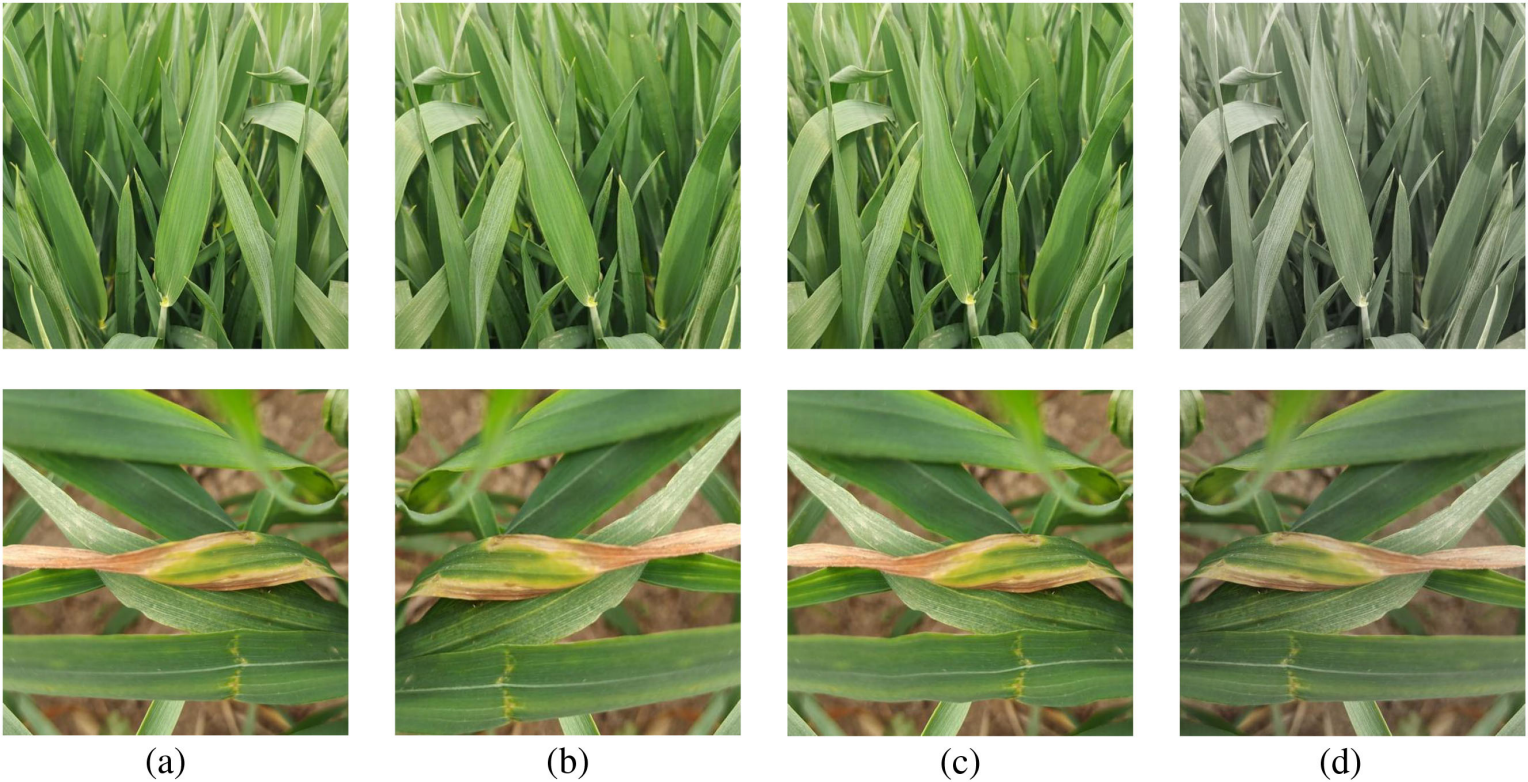
Samples of image augmentation of WL-Disease dataset. **(a)** Original image, **(b)** Left-right flip, **(c)** Distortion, **(d)** Color enhancement.

To facilitate model training, all disease images were uniformly resized to 500 × 500 pixels and converted to JPG format. Data augmentation techniques enhance the effectiveness of neural networks by increasing both the heterogeneity and volume of training data, thereby improving generalization capabilities. Throughout the experiment, due to the limited number of samples per class in the dataset, we applied 10-fold offline data augmentation to mitigate overfitting to specific subsets and improve the model’s stability and accuracy in practical applications. This process included random rotation, 45-degree skew, 10-degree shear operations, 5-strength distortion processing, 50% probability of left-right flip, and color enhancement to expand the training set. **Figure 2** shows the comparison before and after image augmentation.

### 3.2 Problem formulation

Currently, the task of wheat leaf disease identification aims to assign the correct label from a predefined set of categories to an image, achieving precise classification and recognition. A common research approach involves utilizing deep learning algorithms to extract features of wheat leaf diseases and perform recognition. In contrast, this study adopts a methodology that incorporates a deformable prototypical part network with contrastive learning, aiming to achieve interpretable and accurate recognition of wheat leaf diseases. Given a leaf disease image x, its corresponding category label is y ∈ {0*,…,c,…,C*}. The model learns a mapping function ℱ:ℱ(x) → $\hat{\text{y}}$ capable of predicting the category to which the given image x belongs, where $\hat{\text{y}}$ is the probability that the wheat leaf disease image x belongs to its corresponding category. The objective of this research is to optimize the mapping function ℱ to maximize the predicted probability. Meanwhile, the method automatically identifies the affected regions of wheat leaf diseases, providing interpretable evidence for the final classification results.

### 3.3 CDPNet network architecture

**Figure 3:**
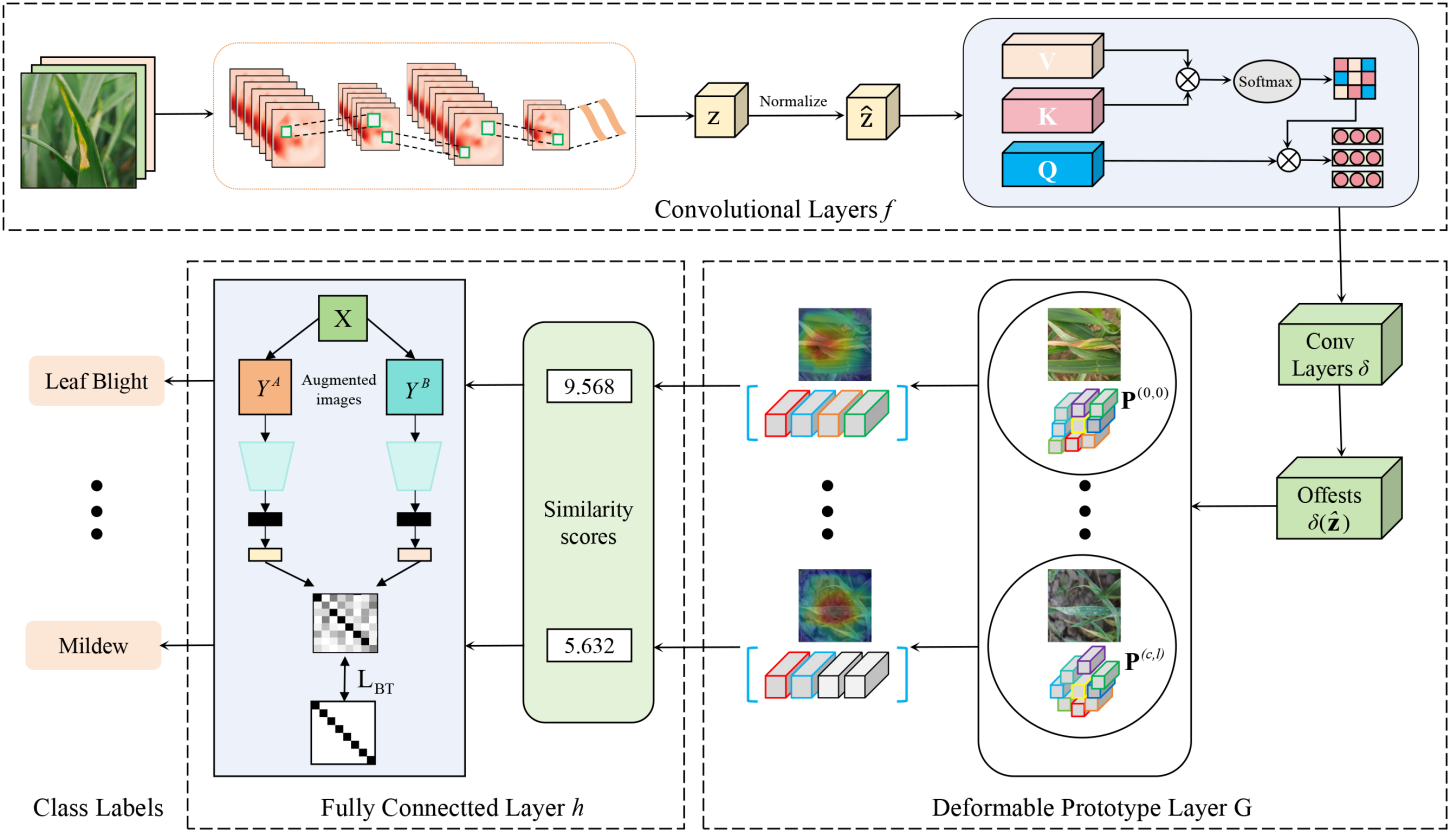
Visualization of the architecture of the proposed CDPNet.

In this section, we provide a detailed description of the architecture of the proposed interpretable wheat leaf disease recognition model based on a deformable prototypical part network and contrastive learning, which is visualized in **Figure 3**. CDPNet primarily consists of convolutional layers f, a deformable prototype layer G, and a fully connected last layer *h*. Given an input image x ∈ *X*, the convolutional layers *f* first extract a meaningful image representation $Z=f(\text{x})\in {\mathbb{R}}^{H\times W\times C}$ (with height *H*, width *W*, and number of channels *C*). Second, for each prototype, the deformable prototype layer G computes a similarity matrix ${\text{M}}_{{\text{p}}_{i}}^{\text{x}}\in {\mathbb{R}}^{H\times W}$ between the convolutional feature maps **Z** and a learnable latent prototype feature representation $P^{\left(c,t\right)}\in {\mathbb{R}}^{1\times 1\times C}$ (the *t*-th prototype of class *c*). The similarity maps contain positive scores indicating where and to what extent prototypes are present in an image. CDPNet uses the highest value of the similarity map as the final similarity score between $P^{\left(c,t\right)}$ and x, indicating how strong the prototype $P^{\left(c,t\right)}$ is present in x. Finally, the similarity scores from the deformable prototype layer G are aggregated in the fully connected layer *h* to generate the final classification logits. These logits are normalized using the softmax function to obtain the predicted probability distribution of disease categories. In addition, to facilitate the visualization of prototypes as specific prototypical parts of a sample, the learned prototypes are substituted with the closest feature representation from authentic training images, thereby ensuring interpretability.

#### 3.3.1 Convolutional layer

The role of the convolutional layers extract information from the input image, which is referred to as image features. These features are manifested through combinations or individual contributions of each pixel within the image, such as texture and color characteristics. Through the convolutional layers, local regional feature extraction of wheat leaf disease images can be achieved, generating the original feature representation of the image. Specifically, the convolutional layers *f* borrow the convolutional layers from classical models (such as VGG19, ResNet152, DenseNet161, etc.), and then two additional 1 × 1 convolutional layers intended to modify the number of channels present in the top-level feature maps. Meanwhile, we use ReLU as the activation function for all convolutional layers, except for the last layer, which employs the sigmoid activation function. Equation 1 converts the input image x into a feature vector.

(1) $$[z_{1},\ldots ,z_{i},\ldots ,z_{m}]=\text{Conv}2\text{D}(\text{x})\in {\mathbb{R}}^{W\times H\times C}$$

**Figure 4:**
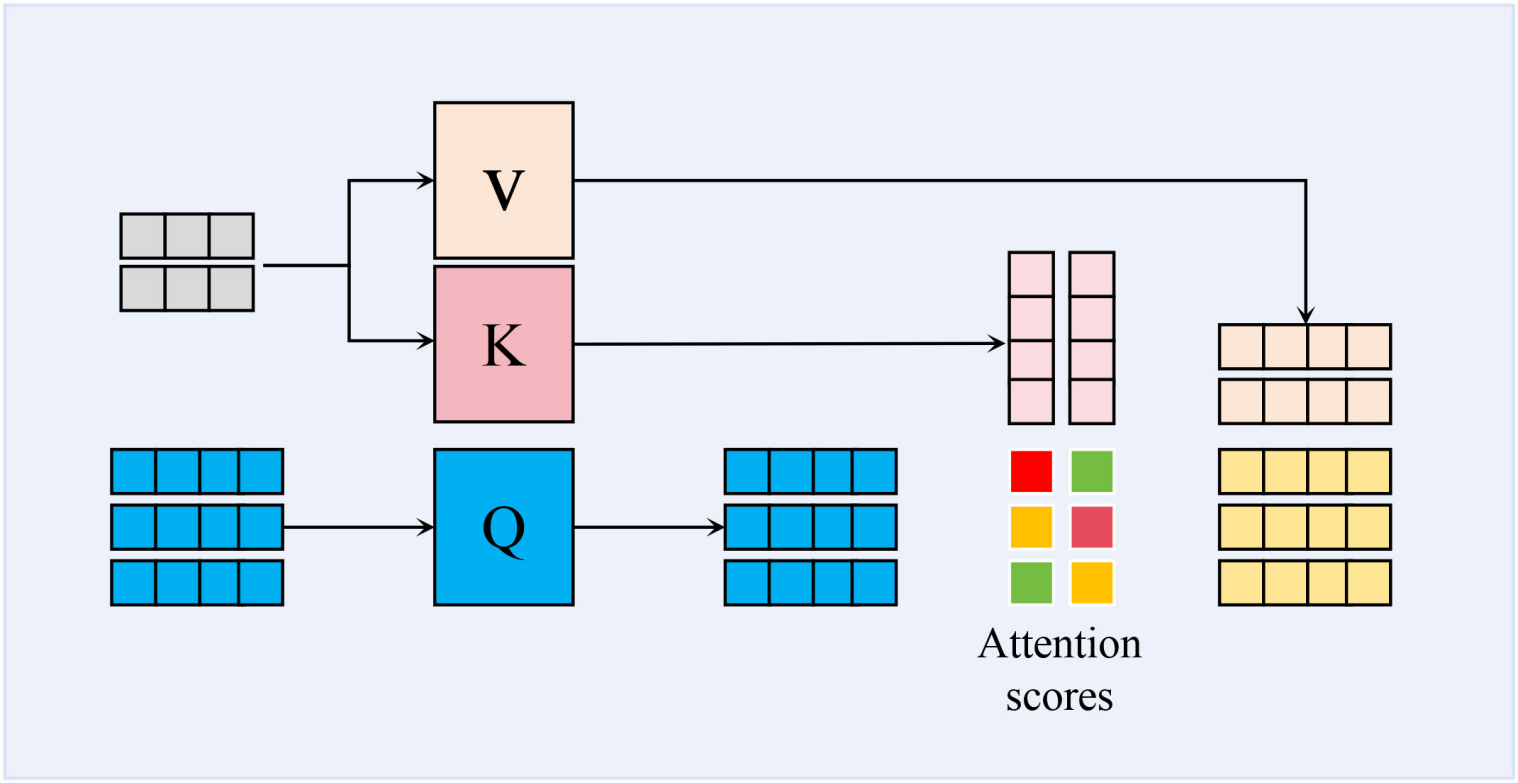
Schematic representation of cross attention mechanism.

To effectively distinguish the target regions of wheat leaf diseases from complex backgrounds, our core method is to employ a CA mechanism, as shown in **Figure 4**. CA mechanism enables the model to dynamically construct cross-modal feature correlation matrices, allowing it to adaptively focus on key discriminative features such as lesion textures and color distortions. It also facilitates a more comprehensive integration of contextual information from multiple sources, consequently boosting both the precision and the generalization performance of the recognition task. Firstly, the correlation scores indicating the similarity between the query and keys are determined by calculating the dot product of the query **Q** and keys **K**. Secondly, these similarities are transformed into a probability distribution using the softmax function, representing the attention weights of the query with respect to each key. These attention weights are then applied to the values **V**, ultimately resulting in the output vector. Mathematically, the formula for cross-attention is presented in Equation 2:

(2) $$\text{CrossAttention}(Q,K,V)=\text{softmax }(\frac{QK^{\text{T}}}{\sqrt{d_{k}}})$$

where, $QK^{T}$ represents the dot product of the query and the key, indicating the similarity between the two sequences at different positions; *d_k_* is the dimension of the key, which serves as a scaling factor to prevent excessively large numerical values.

#### 3.3.2 Deformable prototype layer

The fundamental idea behind the deformable prototype layer G is to find highly interpretable (i.e., representative) deformable prototypes by calculating the similarity scores s between the convolutional feature maps **Z** of a test image x and the prototypes **P**. Each part of these prototypes corresponds to key regions that influence the model’s decision-making processes, and these regions could be visualized. For a CDPNet, the *L*^2^-length of all prototype parts $P_{m,n}^{\left(c,t\right)}$ of all deformable prototypes $P^{\left(c,t\right)}$ is the same. Furthermore, at the spatial location (a,b) of each image feature tensor $\hat{z}$, the corresponding vectors also possess are of equal *L*^2^-length, as shown in Equations 3 and 4.

(3) $$∥P_{m,n}^{\left(c,t\right)}{∥}_{2}=r=\frac{1}{\sqrt{\rho }},$$

(4) $$∥{\hat{z}}_{a,b}{∥}_{2}=r=\frac{1}{\sqrt{\rho }}$$

Then, the formula for calculating the similarity of deformable prototypes $P^{\left(c,t\right)}$ and the image feature tensor $\hat{z}$ defined as shown in Equation 5.

(5) $$G{\left(\hat{z}\right)}_{a,b}^{\left(c,t\right)}=\sum_{m}\sum_{n}P_{m,n}^{\left(c,t\right)}\cdot {\hat{z}}_{a+m,b+n}$$

In order to facilitate the deformation of a deformable prototype $P^{\left(c,t\right)}$, it has been proposed that offsets δ (2D vector) be introduced, thereby enabling each constituent part $P_{m.n}^{\left(c,t\right)}$ of the prototype to migrate in relation to the spatial location (a, b) with respect to the image feature tensor $\hat{z}$ when the prototype is applied. Mathematically, the formula for calculating the similarity of the prototype is defined as shown in Equation 6.

(6) $$G{\left(\hat{z}\right)}_{a,b}^{\left(c,t\right)}=\sum_{m}\sum_{n}P_{m,n}^{\left(c,t\right)}\cdot {\hat{z}}_{a+m+{\text{Δ}}_{1},b+n+{\text{Δ}}_{2}}$$

The maximum similarity with respect to an arbitrary set of positions is given by the following definition.

(7) $$G{\left(\hat{z}\right)}^{\left(c,t\right)}=\underset{a,b}{\max}G{\left(\hat{z}\right)}_{a,b}^{\left(c,t\right)}$$

**Figure 5:**
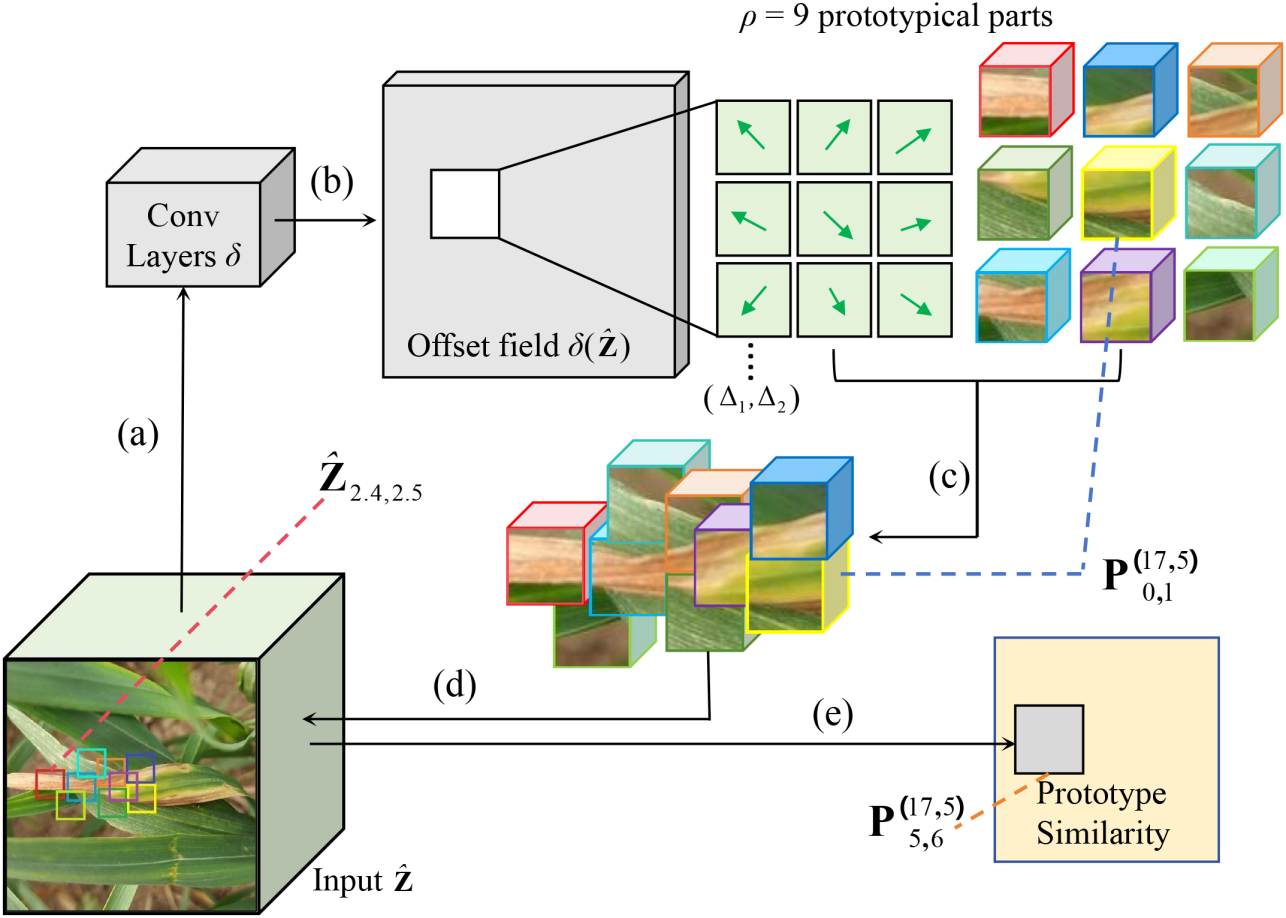
CPNet is applied to the latent representation of the Leaf Blight. **(a)** Put the input features into the offset prediction function to generate **(b)** an offset field. Then, **(c)** adjust the spatial position of each prototypical part using these offsets, **(d)** compare the adjusted parts with the input, and **(e)** calculate the prototype similarity.

**Figure 5** shows the operational process of the deformable prototypes. The input $\hat{z}$ undergoes processing by the offset prediction function δ, resulting in (b) a grid of offset values. Subsequently, these offsets are utilized to (c) modify the spatial positions of each prototypical part. After this adjustment, the updated prototypical parts are (d) aligned with the input to (e) compute the prototype similarity in accordance with Equation 6.

#### 3.3.3 Fully connected layer

The fully connected layer integrates and abstracts the features learned from the preceding layers to facilitate the execution of classification or regression tasks. It performs a linear transformation on the input data using a weight matrix and a bias vector. In the CDPNet model, the fully connected layer multiplies the similarity scores generated by the deformable prototype layer by the weight matrix **W** in the fully connected layer. The result is then feeds the result into the Softmax layer for normalization. Finally, it generates a prediction result for the given leaf disease and pest image. The prediction of the leaf disease image at this point is calculated as shown in Equation 8.

(8) $$\hat{y}=\text{Softmax}(R_{zg}W_{\text{h}}+\text{b})$$

where, $W_{h}\in {\mathbb{R}}^{d\times c}$ is the parameter matrix, represents the image features, b is the bias term, and $\hat{y}=[{\hat{\text{y}}}_{0},\ldots ,{\hat{\text{y}}}_{c},\ldots ,{\hat{\text{y}}}_{C}]$, ${\hat{\text{y}}}_{c}$ denotes the predicted probability that the input image belongs to the *c*-th class. Therefore, given an image x, a novel form of cross-entropy is employed: the margin-subtracted cross-entropy. The formula is shown in Equation 9.

(9) $$C_{ce}(\theta )=\sum_{i=1}^{N}-\text{log }\frac{\exp\;(\sum_{c,t}W_{h}^{\left((c,t),y^{\left(i\right)}\right)}G^{\left(-\right)}{\left(i\right)}^{\left(c,t\right)})}{\sum_{c^{\prime }}\exp\;(\sum_{c,t}W_{h}^{\left((c,t),c^{\prime }\right)}G^{\left(-\right)}{\left(i\right)}^{\left(c,t\right)})}$$

where, θ  represents the parameters that need to be learned, and $W_{h}^{\left((c,t),c^{\prime }\right)}$ denotes the connections between the deformable prototypes $P^{\left(c,t\right)}$ and the last layer responsible for computing similarity with the *c* ' classes.

#### 3.3.4 Model learning

**Figure 6:**
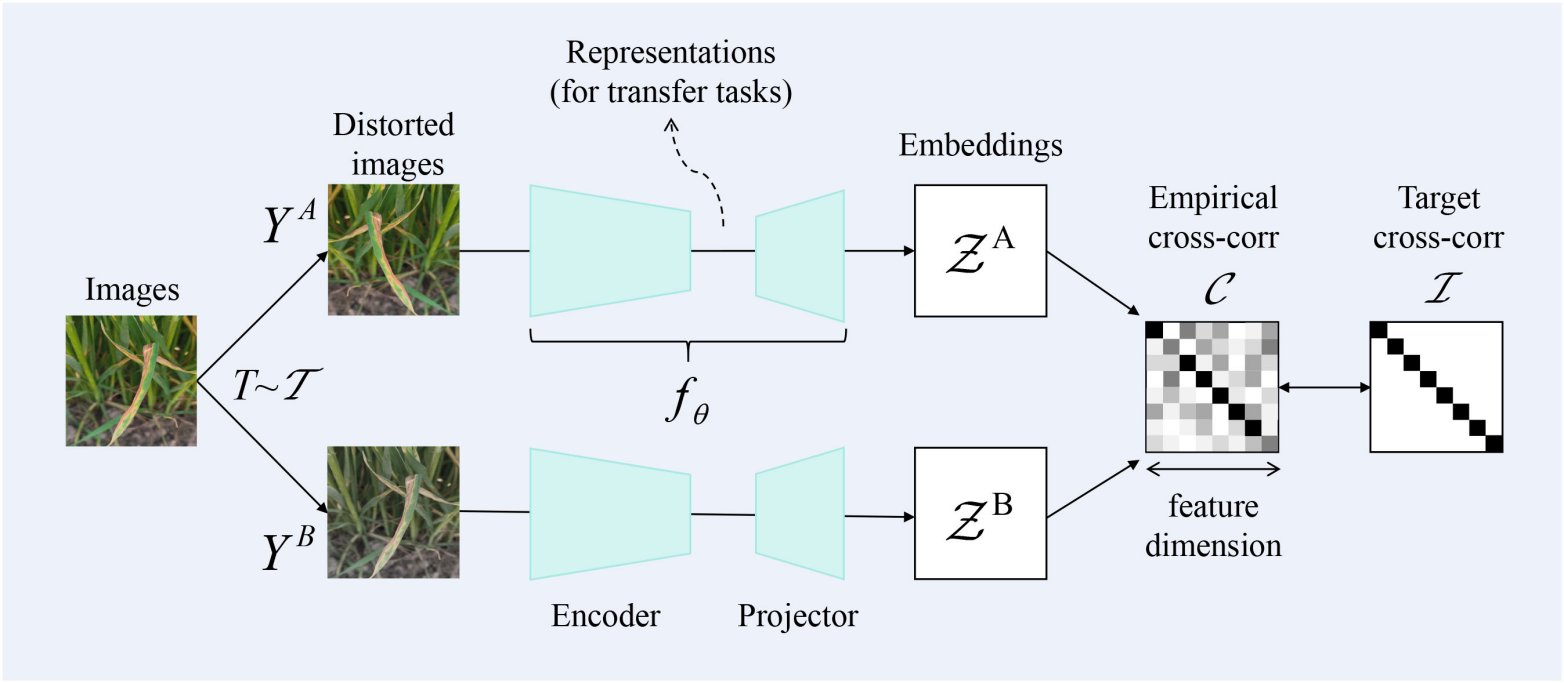
Schematic representation of Barlow Twins.

As deep learning progresses, approaches for identifying wheat leaf diseases harness deep networks to automatically learn features; however, these methods heavily depend on the availability of a substantial volume of training data. To address the limited availability of wheat image data, we have introduced a self-supervised contrastive learning approach to tackle the challenge of recognition with limited samples. Specifically, **Figure 6** shows that we used the Barlow Twins in contrastive learning to conduct feature learning between samples. Barlow Twins represents a self-supervised learning approach for representation learning, stemming from the groundbreaking ideas of the JPT team. Its core lies in minimizing the covariance distance between twin networks, enabling their learned features to be as independent as possible while maintaining similarity. This approach not only enhances the efficiency of the model but also achieves favorable pre-training results even with scarce data.

Barlow Twins is a self-supervised learning method rooted in information theory, with the objective of reducing redundancy among neurons. This approach mandates that neurons remain invariant to data augmentations while being independent of one another. During actual training, the parameters of the neural network are adjusted through backpropagation to maximize the diagonal elements of the cross-correlation matrix and minimize the off-diagonal elements — approaching an identity matrix — thereby achieving the aforementioned goal. It is calculated as shown in Equation 10.

(10) $$L_{BT}=\sum_{i}{\left(1-C_{ii}\right)}^{2}+\lambda \sum_{i}\sum_{j\neq i}C_{ij}^{2}$$

where λ is a positive constant trading off the importance of the first and second terms of the loss, $\;\sum_{i}{\left(1-C_{ii}\right)}^{2}$ is an invariance term (diagonal or identity term) designed to direct neurons to produce the same output under different augmentations, $\sum_{i}\sum_{j\neq i}C_{ij}^{2}$ is a redundancy reduction term (off-diagonal term) intended to make each neuron produce a different output.

(11) $$C_{ij}=\frac{\sum_{b}Z_{b,i}^{A}Z_{b,j}^{B}}{\sqrt{\sum_{b}{\left(Z_{b,i}^{A}\right)}^{2}\sqrt{\sum_{b}{\left(Z_{b,j}^{B}\right)}^{2}}}}$$

where, *b* denotes the index of the batch, while *i* and *j* represent the feature dimensions of the network’s output (i.e., they correspond to the values in the *i*-th and *j*-th dimensions of two vectors within the current batch). $C_{ij}$ is the element value at the *i*-th row and *j*-th column of matrix C. It is equal to the sum of the products of the *i*-th dimension of the augmented feature vector $Z^{\text{A}}$ and the *j*-th dimension of the augmented feature vector $Z^{\text{B}}$ for different pairs within the batch. The summation is primarily carried out over the current batch size. Matrix C is a square matrix, and its dimensions correspond to the output dimension of the network (assuming each embedding dimension output by the network is D, then the dimensions of square matrix C are D×D). The values of matrix C range between -1 (indicating perfect negative correlation) and 1 (indicating perfect positive correlation).

In order to discover a meaningful feature space in which the image features belonging to class c are found to cluster around the prototypes of the same class while being segregated from features of other classes within a hypersphere, CDPNet employs Stochastic Gradient Descent (SGD) to perform optimization on the features of the convolutional layer *f* and the deformable prototype layer G. In this process, SGD incorporates both cluster and separation losses and adjusts the angular space. These two losses are defined as shown in Equations 11 and 12.

(12) $$C_{clst}=-\frac{1}{N}\sum_{i=1}^{N}\underset{P^{\left(c,t\right)}:c=y^{\left(i\right)}}{\max}G{\left({\hat{z}}^{\left(i\right)}\right)}^{\left(c,t\right)}$$

(13) $$S_{\text{sep}}=\frac{1}{N}\sum_{i=1}^{N}\overset{N}{\underset{P^{\left(c,t\right)}:c\neq y^{\left(i\right)}}{\max}}G{\left({\hat{z}}^{i}\right)}^{\left(c,t\right)}$$

where, *N* represents the total number of inputs, ${\hat{z}}^{\left(i\right)}$ denotes the normalized and scaled image feature tensor of input i at each spatial location, ${\text{y}}^{\left(i\right)}$ is the label corresponding to input ${\text{x}}^{\left(i\right)}$, and all other values are consistent with the definitions provided in the preceding context.

Although the subtraction margin encourages separation among categories, it does not promote diversity among intra-class prototypes or within prototype parts within a prototype. Specifically, deformations without further regularization often lead to redundancy among prototype parts within a prototype. To mitigate this issue, we prevent this behavior by introducing an orthogonality loss among prototype parts. Its formula is shown in Equation 14.

(14) $$O_{\text{ortho}}=\sum_{c}\|P^{\left(c\right)}P^{(c)⊤}-r^{2}I^{\left(\rho L\right)}{\|}_{F}^{2}$$

where L is the number of deformable prototypes in class *c*, ρL  represents the total number of prototype parts across all prototypes in class *c*, $I^{\left(\rho L\right)}$ is the ρL×ρL identity matrix, and $P^{\left(c\right)}\in {\mathbb{R}}^{\rho L\times d}$ is a matrix where each prototype part of every prototype in class *c* is arranged as a row.

Finally, the overall loss function during the CDPNet training process is formulated as shown in Equation 15.

(15) $$L_{total}=C_{ce}(\theta )+\lambda_{1}C_{clst}+\lambda_{2}S_{\text{sep}}+\lambda_{3}O_{\text{ortho}}+\lambda_{4}L_{BT}$$

## 4 Results and analysis

### 4.1 Experimental setup

In this study, the PyTorch framework was utilized. PyTorch is an open-source library designed for deep learning tasks, offering a concise, elegant, efficient, and rapid framework that serves as a deep learning research platform providing maximum flexibility and speed. The experimental environment and parameters used in this study are detailed in **Table 2**.

**Table 2:** Test system environment configuration.

System environment	Configuration
Operating system	Ubuntu 18.04
GPU	V100-32GB(32GB)
CPU	10 vCPU Intel Xeon Processor (Skylake, IBRS)
Pytorch	PyTorch 1.8.0
Python	Python 3.8
Batch size	32
Epoch	50

### 4.2 Evaluation metrics

We validated the model’s effectiveness on the test set using standard classification performance metrics. These metrics include accuracy, precision, recall, F1-score, and AUC. Their mathematical expressions are as shown in Equations 16–21. All samples were categorized into four groups based on the differences between the true and predicted classes: true positives (TP), false positives (FP), true negatives (TN), and false negatives (FN).

(16) $$Accuracy=\frac{TP+TN}{TP+TN+FP+FN}$$

(17) $$P\text{re}cisio\text{n}=\frac{TP}{TP+FP}$$

(18) $$R\text{e}call=\frac{TP}{TP+FN}$$

(19) $$F1-Score=2*\frac{\text{Pr}ecision\;*\;Recall}{\text{Pr}ecision+Recall}$$

(20) $$TPR=\frac{TP}{TP+FN}$$

(21) $$FPR=\frac{FP}{FP+TN}$$

In addition, we employed the confusion matrix and Receiver Operating Characteristic (ROC) curve to evaluate the model’s performance. The confusion matrix and ROC curve indicate the model’s credibility. The higher the ROC curve is positioned in the top-left corner, the better the model’s performance. Meanwhile, we utilized CDPNet to visualize the prototype image classification activation maps and similarities, aiming to uncover the critical factors underlying the model’s classification decisions and assist researchers in understanding the basis for the model’s final classifications.

### 4.3 Experimental results and comparative analysis

#### 4.3.1 Performance evaluation of different data augmentation methods

**Table 3** shows the results of experiments conducted using the CDPNet-DenseNet161 model with various data augmentation methods. Six distinct data augmentation schemes were generated by combining different techniques. Scheme 1 involved inputting the original image into the model after normalization (resizing to 224×224×3), resulting in a classification accuracy of 92.25%. Subsequently, the introduction of various data augmentation methods, including skew, shear, distortion, left-right flipping, and color enhancement, to Scheme 1 led to an improvement in model accuracy. Among the augmentation techniques tested, color enhancement produced the most favorable results. The results indicate that Scheme 6 achieved the highest accuracy (95.83%), establishing it as the optimal data augmentation scheme.

**Table 3:** Comparison of experimental results of different models on wheat leaf disease dataset.

No.	Data augmentation methods	Accuracy (%)
1	Resize(224,224)	92.25
2	Resize(224,224)+skew	92.56
3	Resize(224,224)+skew+shear	93.36
4	Resize(224,224)+skew+shear+distortion	93.82
5	Resize(224,224)+skew+shear+distortion+left-right flipping	94.76
6	Resize(224,224)+skew+shear+distortion+left-right flipping+color enhancement	95.83

#### 4.3.2 Model performance comparisons

**Figure 7:**
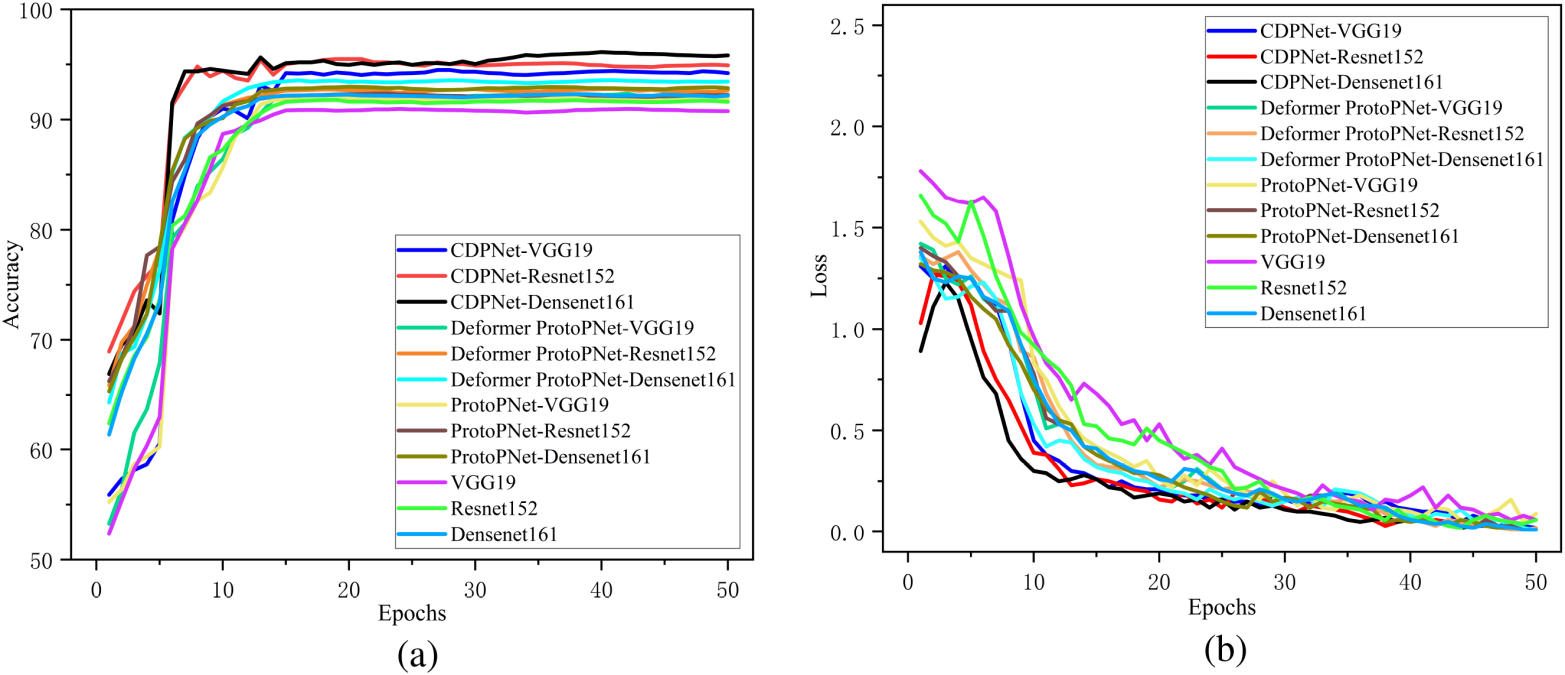
Comparison of the variation curves for loss values and accuracy across different models. **(a)** Accuracy curve, **(b)** Loss curve.

**Figure 8:**
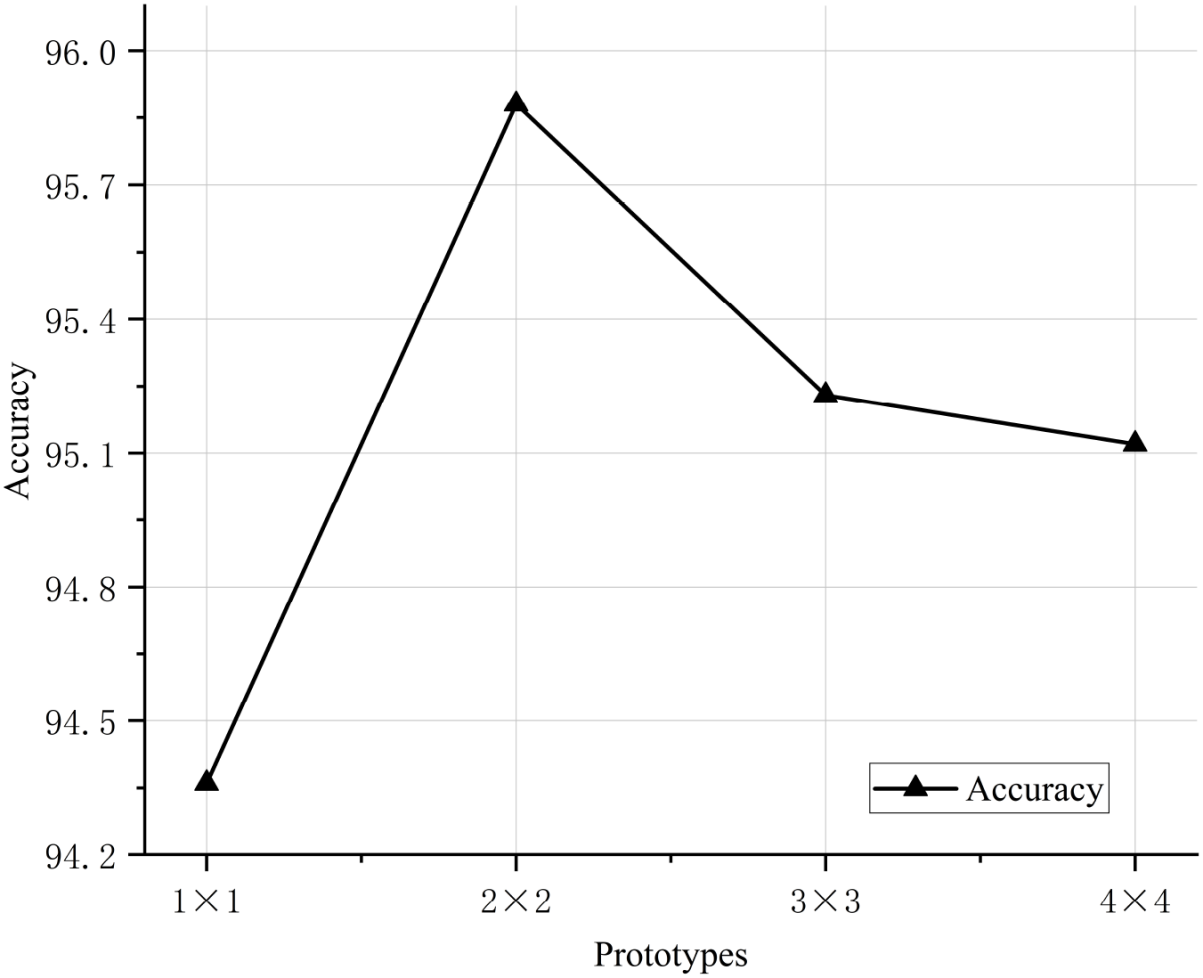
Impact of CDPNet-DenseNet161 to the number of prototypes selected on accuracy.

**Figure 9:**
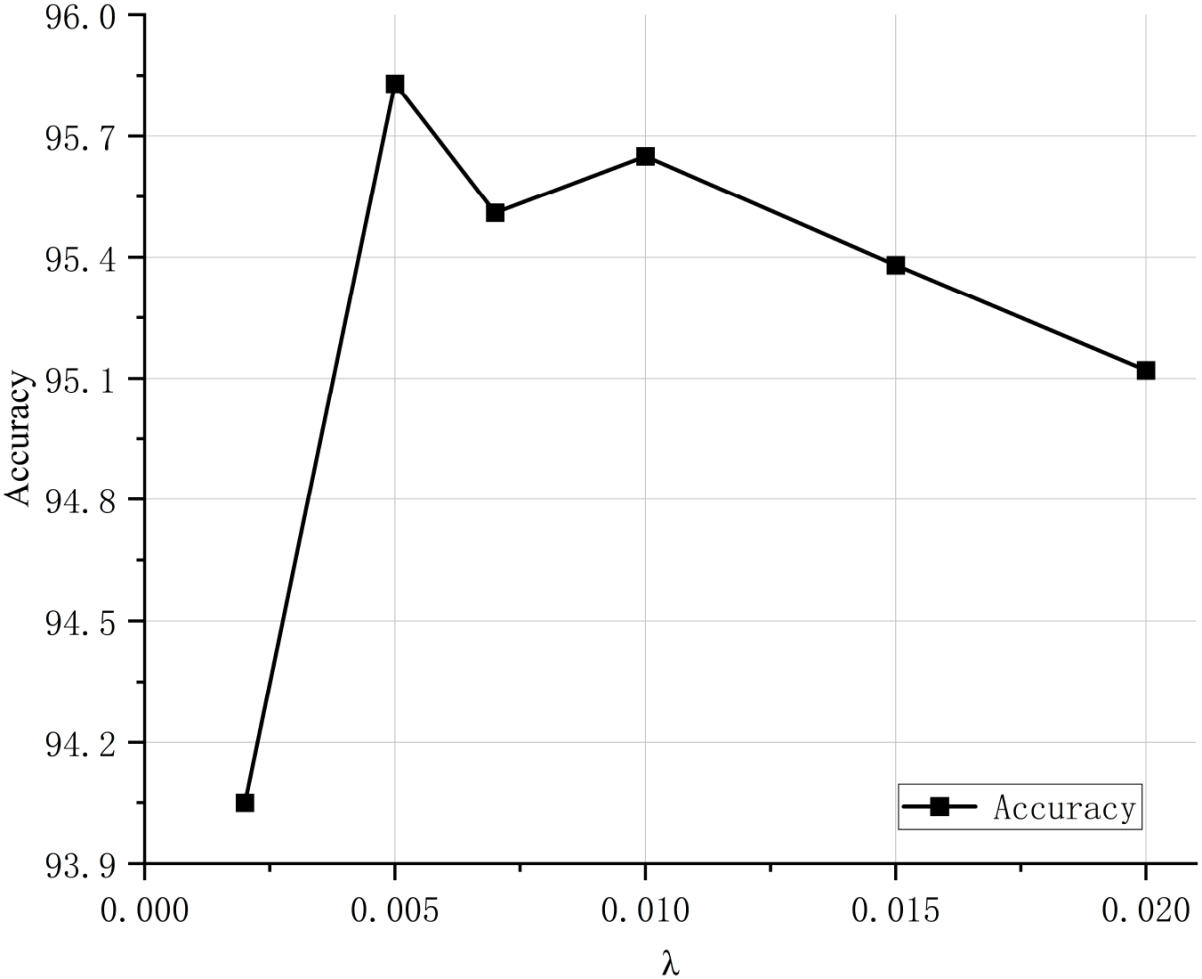
Sensitivity of the Barlow Twins component in CDPNet-DenseNet161 to hyperparameter *λ*.

To validate the classification performance of the proposed CDPNet model for wheat leaf diseases, comparative experiments were conducted under identical conditions using the WL-Disease dataset, comparing CDPNet with VGG19, ResNet152, DenseNet161, ProtoPNet, and Deformer ProtoPNet models. The comparative results for each model are shown in **Table 4**. **Figure 7** shows the loss value and accuracy comparison curves during the training phase for different model. **Table 4** shows that CDPNet outperforms 416 the other models on the WL-Disease dataset with statistical significance. Compared to DenseNet161, the baseline model, CDPNet achieves an accuracy of 95.83%, representing improvements of 2.35%, 3.02%, and 3.65% over Deformer ProtoPNet, ProtoPNet, and DenseNet161, respectively. **Figure 7** shows that 419 throughout the entire training process, the CDPNet model consistently outperforms the other four models in both accuracy and loss values, further validating its faster convergence speed. In **Figure 8**, we explore the effect of varying the prototype count per class on classification performance. CDPNet achieves optimal classification accuracy (95.88%) with 2×2 prototypes configuration, outperforming models with other prototype settings. Therefore, 2×2 prototypes was adopted for all subsequent experiments. **Figure 9** presents the sensitivity analysis of CDPNet, based on the DenseNet161 backbone, with respect to its key components. **Figure 9** demonstrates the sensitivity of the Barlow Twins component to the hyperparameter λ, which governs the trade-off between invariance and information density in the embedding space. The results indicate that the Barlow Twins are relatively insensitive to this hyperparameter.

**Table 4:** Comparison of experimental results of different models on wheat leaf disease dataset.

Model	Accuracy (%)	Precision (%)	Recall (%)	F1 score (%)	AUC (%)
VGG19	90.38	90.67	90.89	90.50	97.69
ResNet152	91.61	91.36	91.08	91.03	97.92
DenseNet161	92.18	91.86	91.63	91.76	98.16
ProtoPNet-VGG19	91.85	91.38	91.45	91.35	98.02
ProtoPNe-ResNet152	92.16	91.82	91.52	91.66	98.08
ProtoPNet-DenseNet161	92.81	92.45	92.63	92.53	98.33
Deformer ProtoPNet-VGG19	92.15	91.83	91.52	91.65	98.12
Deformer ProtoPNe-ResNet152	92.63	92.27	92.11	92.19	98.25
Deformer ProtoPNet-DenseNet161	93.48	93.13	92.89	92.99	98.52
CDPNet-VGG19	$94.22^{c}$	$93.72^{c}$	$93.97^{c}$	$93.77^{c}$	$99.16^{c}$
CDPNet-ResNet152	$94.89^{c}$	$94.21^{c}$	$94.47^{c}$	$94.29^{c}$	$99.38^{c}$
CDPNet-DenseNet161	$95.83^{c}$	$95.32^{c}$	$95.07^{c}$	$95.13^{c}$	$99.45^{c}$

*^c^*Denotes the test of statistical significance *p <* 0.001.

**Figure 10:**
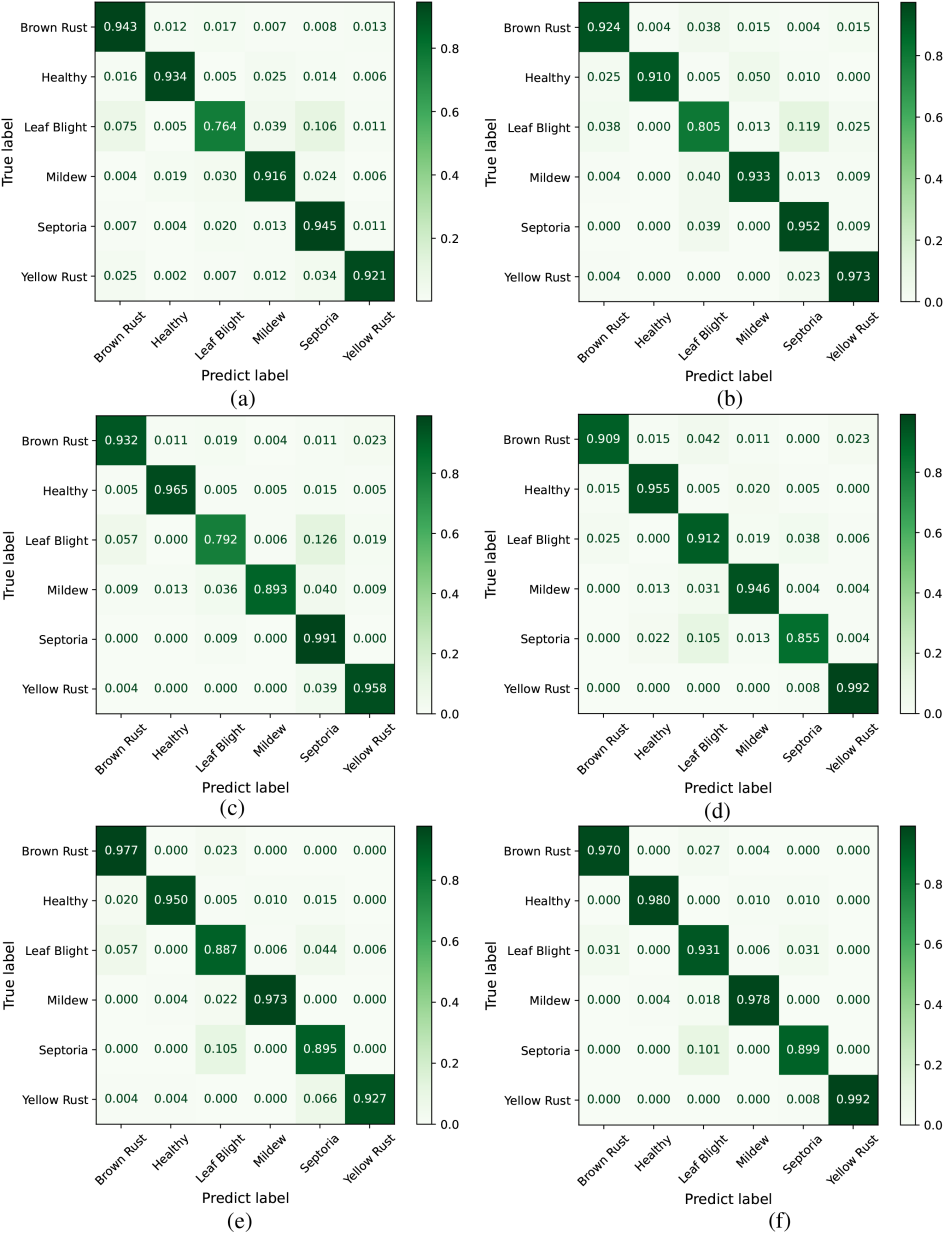
Comparison of confusion matrices across different models. **(a)** VGG19, **(b)** ResNet152, **(c)** DenseNet161, **(d)** ProtoPNet-DenseNet161, **(e)** Deformer ProtoPNet-DenseNet161, **(f)** CDPNetDenseNet161.

**Figure 10** shows a confusion matrix that intuitively represents the relationship between predicted results and actual class labels. This illustrates the effectiveness of the model’s classification capabilities. In **Figure 10a**, Leaf Blight exhibits the lowest classification accuracy (76.4%), with 10.6% of test images being misclassified as Septoria and 7.5% misclassified as Brown Rust. In **Figure 10d**, Septoria has the lowest classification accuracy (85.5%), where 10.5% of test images were incorrectly classified as Leaf Blight. This phenomenon stems primarily from two factors: On one hand, Leaf Blight exhibits a dispersed feature distribution within the dataset, lacking distinct clustered patterns that complicate accurate model recognition. On the other hand, Septoria shares highly similar disease characteristics with Leaf Blight, with significant overlaps in visual features such as morphology and coloration, further exacerbating classification challenges. Compared to other models, the deeper colors along the diagonal of CDPNet’s confusion matrix indicate that the majority of classification outcomes are concentrated there. This suggests that the CDPNet model achieves higher recognition accuracy for various diseases, particularly for those with dispersed and easily confused disease regions, such as Leaf Blight and Septoria.

**Figure 11:**
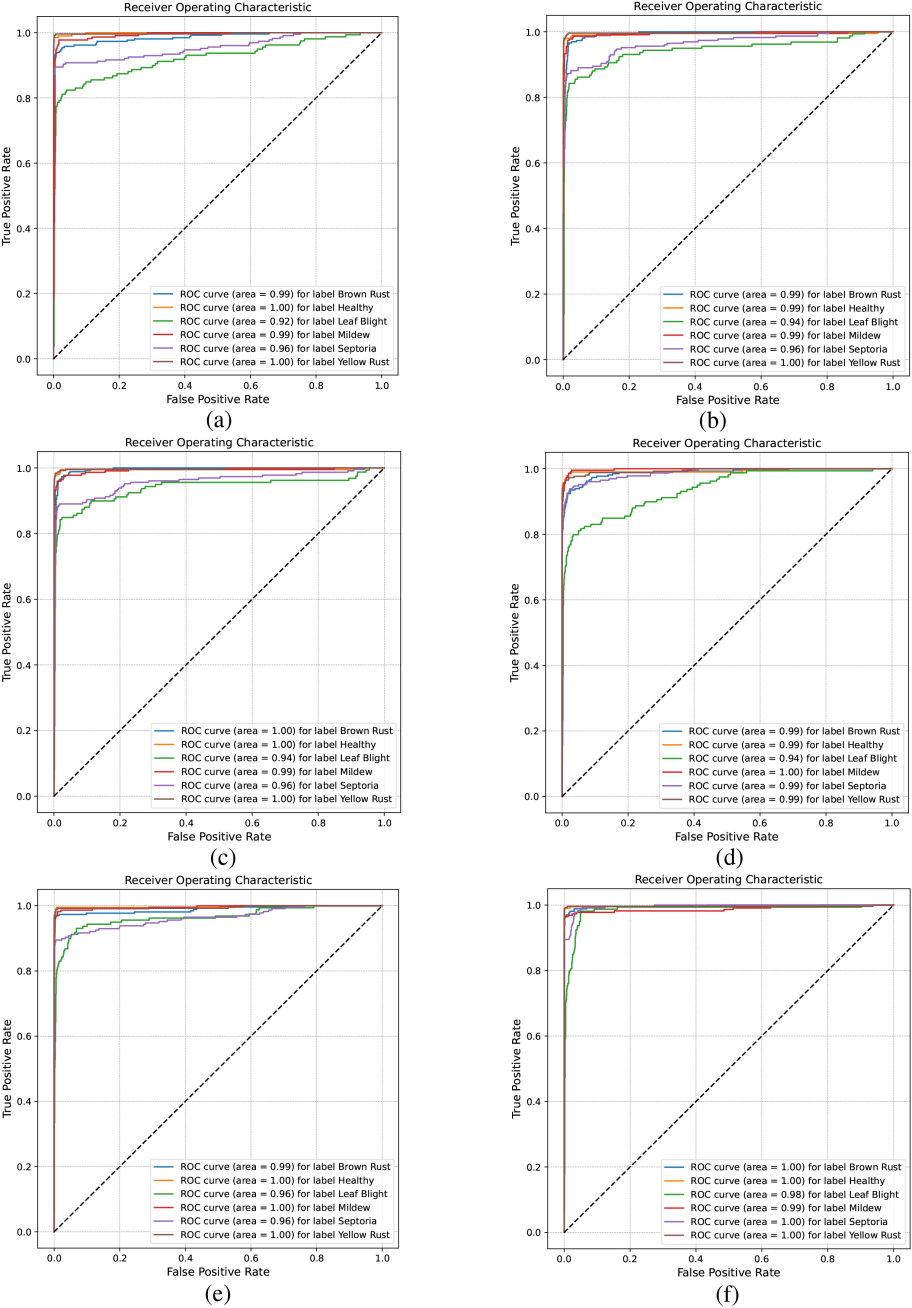
Comparison of ROC across different models. **(a)** VGG19, **(b)** ResNet152, **(c)** DenseNet161, **(d)** ProtoPNet-DenseNet161, **(e)** Deformer ProtoPNet-DenseNet161, **(f)** CDPNet-DenseNet161.

**Figure 12:**
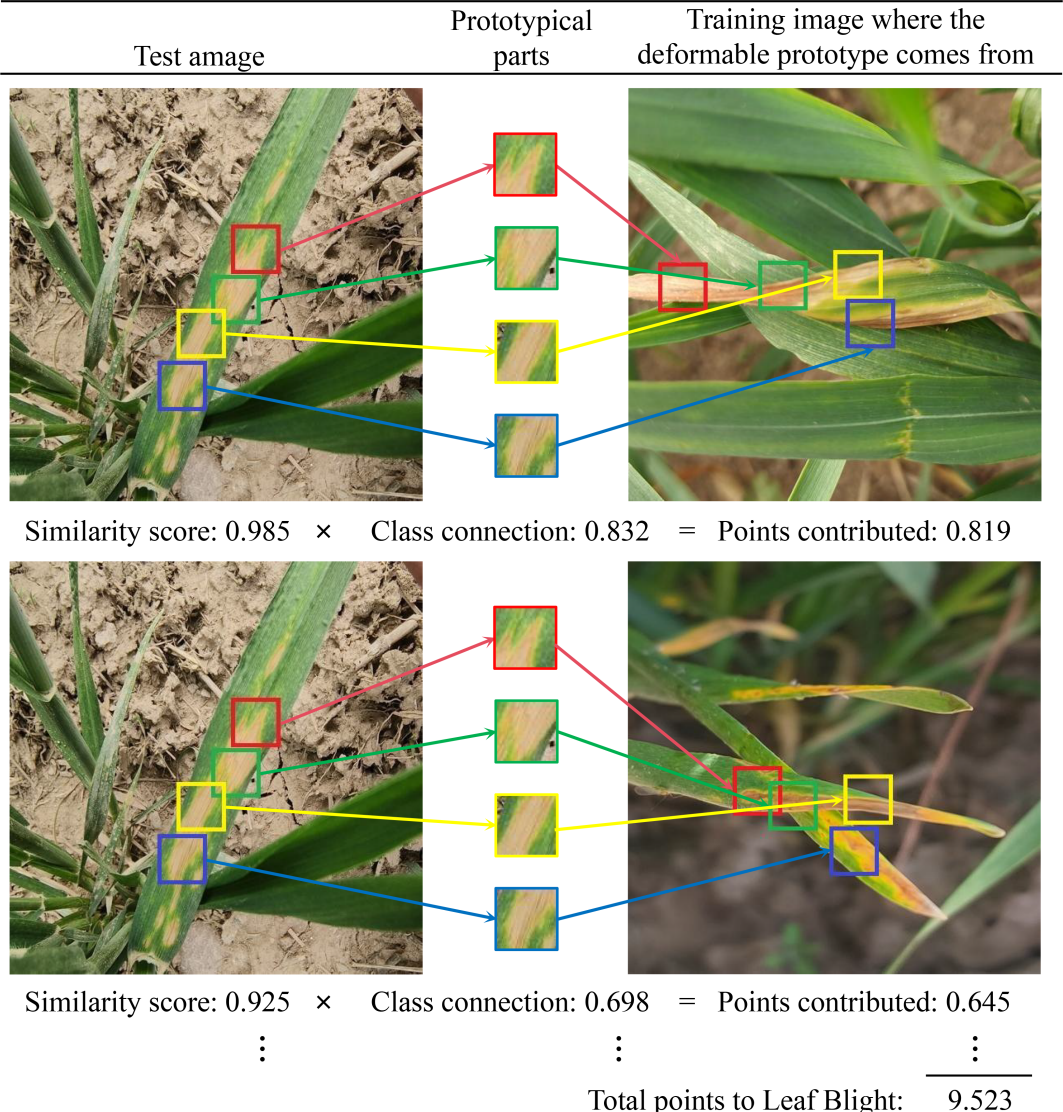
The reasoning process of a CDPNet with 2×2 deformable prototypes.

**Figure 13:**
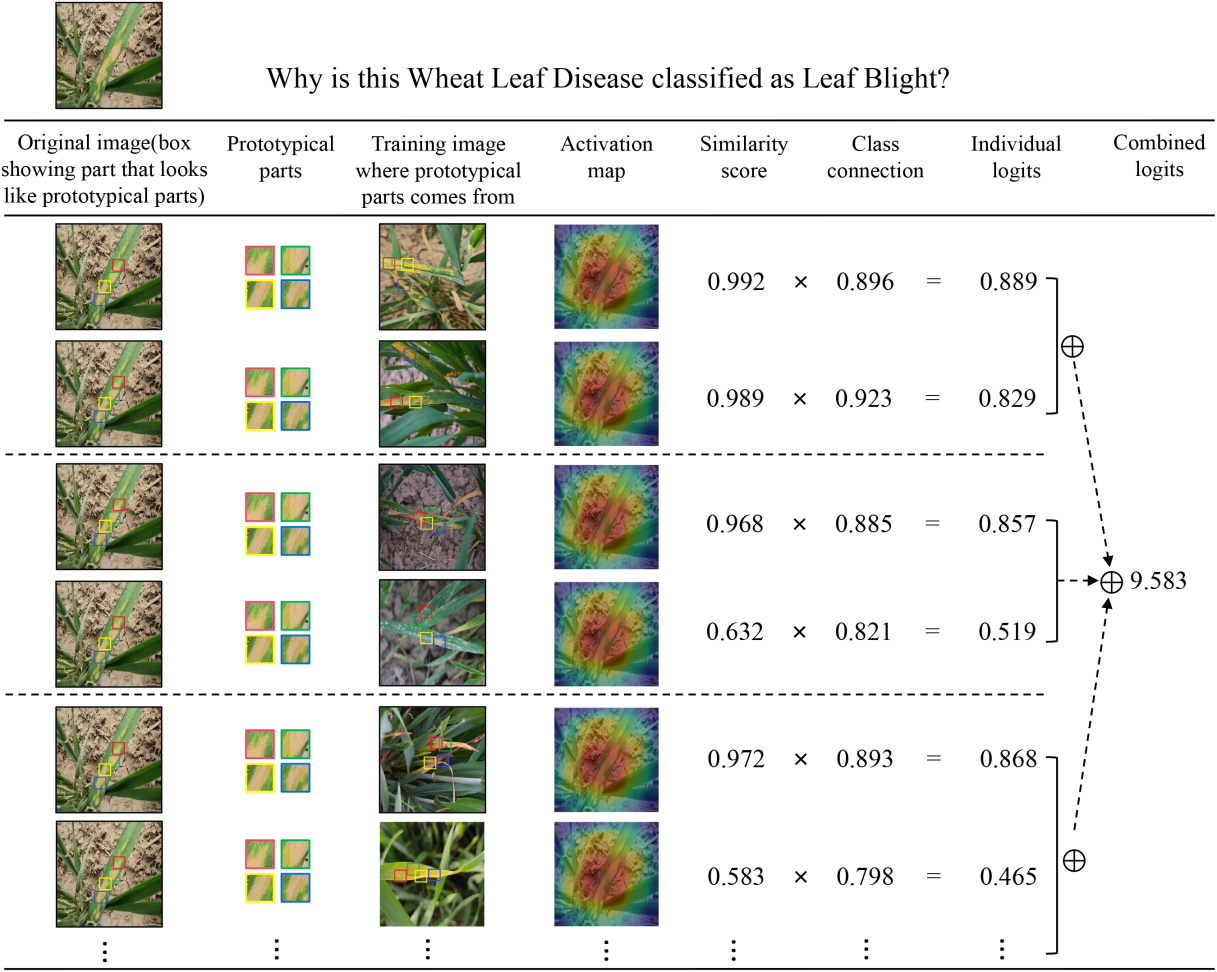
The reasoning process of CDPNet in deciding the species of the wheat leaf blight.

The ROC curve in **Figure 11** helps analyze classification performance across different threshold settings. When comparing the ROC curves of different models, those with a higher AUC indicate better performance. As shown in **Figures 11a–e**, the AUC values for Leaf Blight and Septoria leaf diseases are comparatively low. From a phytopathological perspective, Leaf Blight and Septoria diseases are often misidentified in the field. This is primarily due to their highly similar visual symptoms, including leaf necrosis and the yellow halo resulting from chlorophyll degradation, which makes reliable visual differentiation difficult. In contrast, **Figure 11f** shows that CDPNet demonstrated the highest AUC, achieving superior identification accuracy for these commonly confused diseases. Experimental results indicate that the introduction of the CA mechanism and Barlow twin contrastive learning enabled CDPNet to achieve deeper feature learning for wheat leaf diseases. First, the CA mechanism allows adaptive learning of feature weights across channels, effectively amplifying responses to key disease-related features (e.g., lesion texture, color changes) while suppressing background noise. Second, contrastive learning maximizes similarity between different transformations of the same image while minimizing similarity between different images, thereby optimizing feature relationships across samples and enhancing feature discriminability. As a result, CDPNet improves recognition accuracy for the commonly confused Leaf Blight and Septoria diseases. Moreover, CDPNet’s interpretable outputs (**Figures 12**, **13**) help agronomists distinguish these diseases by highlighting specific visual patterns used by the model (e.g., lesion shape, distribution), potentially revealing features that are challenging for the human eye to discern.

#### 4.3.3 K-fold cross-validation

To further validate the model’s performance stability on the WL-Disease dataset, we employed k-fold cross-validation, processing the dataset sequentially and randomly dividing it into four parts. In each partition, 20% of the data was used as the test set, while the remaining 80% was combined with the other three parts to create a new training set. This approach ensured that each part served as the test set for one partition. We selected DenseNet161 as the baseline model, trained the CDPNet on the training set, validated it on the test set, and recorded the results. **Table 5** displays the results of the 5-fold cross-validation. The WL-Disease dataset achieved an average accuracy of 95.83%, with accuracy fluctuations not exceeding 2% across the cross-validation. The results indicate that CDPNet demonstrates stable performance across different subsets, showcasing strong robustness and excellent generalization ability. The model is not prone to significant performance fluctuations due to changes in data partitioning. This suggests that the model does not overfit to specific subsets but learns general features from the data, exhibiting outstanding generalization performance.

**Table 5:** CDPNet+DenseNet161 test results based on k-fold cross-validation.

No of fold	Accuracy (%)
1-flod	95.22
2-flod	95.56
3-flod	96.13
4-fold	95.89
5-fold	96.36
Average	95.83(± 0.61)

#### 4.3.4 Ablation experiments

To further evaluate the effectiveness of the optimization strategies proposed in this study, ablation experiments were performed. The corresponding results are presented in **Table 6**, which highlights the contribution of each optimization strategy to model performance. Evaluation metrics included accuracy, precision, recall, F1-score on the test set, as well as the number of model parameters. As shown in **Table 6**, the incorporation of the CA mechanism and the contrastive loss function improved the model’s recognition accuracy. Compared with the original Deformer ProtoPNet and using DenseNet161 as the baseline, the CDPNet model, integrating both the CA mechanism and the contrastive loss function, achieved an accuracy of 95.83%, representing an improvement of 2.35%. Furthermore, the precision, recall, F1-score, and AUC improved by 2.22%, 2.18%, 2.14%, and 0.93%, respectively. These findings confirm that the integration of the CA mechanism and the contrastive loss function not only avoided adverse effects but also substantially enhanced the recognition performance of CDPNet.

**Table 6:** CDPNet results of ablation experiment.

Model	Cross Attention	Barlow twin loss	Accuracy (%)	Precision (%)	Recall (%)	F1 score (%)	AUC (%)
Deformer ProtoPNet+VGG19			92.15	91.83	91.52	91.65	98.12
Deformer ProtoPNe+ResNet152			92.63	92.27	92.11	92.19	98.25
Deformer ProtoPNet+DenseNet161			93.48	93.13	92.89	92.99	98.52
Deformer ProtoPNet+VGG19	√		92.85	92.35	92.58	92.45	98.31
Deformer ProtoPNe+ResNet152	√		93.38	92.72	92.97	90.80	98.46
Deformer ProtoPNet+DenseNet161	√		94.13	93.55	93.96	93.62	98.85
Deformer ProtoPNet+VGG19		√	93.64	93.32	93.07	93.13	98.58
Deformer ProtoPNe+ResNet152		√	94.26	93.66	93.62	93.73	99.25
Deformer ProtoPNet+DenseNet161		√	95.25	94.77	95.06	94.83	99.30
Deformer ProtoPNet+VGG19	√	√	94.22	93.72	93.97	93.77	99.16
Deformer ProtoPNe+ResNet152	√	√	94.89	94.21	94.47	94.29	99.38
Deformer ProtoPNet+DenseNet161	√	√	95.83	95.32	95.07	95.13	99.45

“√” indicates that this module has been added.

#### 4.3.5 Experimental comparison of public datasets

To validate the generalization ability of the improved CDPNet model, a series of comparative experiments were performed on the PlantVillage and LWDCD 2020 datasets, alongside our self-built dataset. PlantVillage is an open-source plant disease dataset constructed based on image collection of plant leaves. These images were captured under controlled environmental conditions and cover 14 different species of plant. The dataset comprises approximately 54,305 images, categorized into 38 plant disease classes and 1 background image category. For our model training, we selected image data of three different diseases, such as Apple and Corn diseases, from the PlantVillage dataset. The LWDCD 2020 dataset for wheat diseases consists of nearly 7,000 relatively distinct close-up images of wheat diseases, categorized into 12 classes of common wheat diseases in China based on different disease types. Given that our task is wheat leaf disease identification, we selected five kinds of such diseases for model training. Using DenseNet161 as the baseline model, we trained the CDPNet on the training sets of the three datasets and validated it on the corresponding test sets, recording the validation results. **Table 7** presents the experimental results of the CDPNet model on the three datasets.

**Table 7:** CDPNet performance on public datasets.

Dataset	Accuracy (%)	Precision (%)	Recall (%)	F1 score (%)	AUC (%)
PlantVillage-3	92.55	92.69	92.95	92.68	97.83
LWDCD 2020-5	93.35	92.89	93.61	92.78	98.15
WL-Disease	95.83	95.32	95.07	95.13	99.45

#### 4.3.6 CDPNet interpretability analysis

As an interpretable model, CPDNet not only predicts leaf disease categories but also identifies key affected regions that influence model decisions, enabling explainable image classification and recognition of wheat leaf diseases. **Figure 12** illustrates how CPDNet identifies evidence of leaf blight in the test image by comparing its latent features with each variable prototype within the category (each prototypical part is displayed in the “Prototypical parts” column). As shown in **Figure 13**, when variable prototypes scan the input image, they adaptively adjust their spatial positions. Then, the Prototype similarity scores are computed for each center position using Equation 6. Subsequently, the maximum score across all spatial positions is selected using Equation 7 to generate a single “similarity score” for the prototype. This similarity score is multiplied by the class connection score from the fully connected layer to yield the prototype’s contribution to the classification result. Finally, the contribution scores of all prototypes are summed to obtain the final classification score for the category. **Figures 12**, **13** clearly demonstrate that CPDNet can accurately identify regions most affected by Leaf Blight, facilitating the classification and identification of wheat leaves. As a result, CPDNet’s interpretable output mechanism offers agronomists an intuitive visualization tool, enabling them to focus on specific visual features (e.g., lesion morphology, spatial distribution) and uncover potential diagnostic characteristics that are challenging to detect through traditional visual inspection.

## 5 Conclusion

This work introduces a novel deep learning model with intrinsic interpretability for the identification of wheat leaf diseases. Specifically, we present the CDPNet approach, which identifies key regions influencing model decisions by calculating similarity values between convolutional feature maps and latent prototype feature representations. CDPNet incorporates a CA mechanism to effectively isolate target diseased regions from complex backgrounds, thereby enhancing the model’s feature extraction capabilities. To address the limited availability of wheat leaf disease image data, we employ a self-supervised contrastive learning approach to capture cross-sample features, thereby improving model efficiency. To validate the model’s effectiveness, systematic experiments were conducted using both our self-constructed WL-Disease dataset and two public datasets. The results demonstrate that the proposed CDPNet not only achieves significantly higher accuracy than baseline methods but also provides an interpretable decision-making bases, offering reliable support for practical wheat disease diagnosis in field settings. In summary, the proposed CDPNet model achieves an average accuracy exceeding 92.55% across all three datasets, showcasing its ability to effectively classify and identify diverse crop diseases in real agricultural scenarios.

Future research will focus on developing pre-trained neural network model weights for large-scale plant pest and disease datasets in real-world agricultural settings. This will facilitate the faster convergence of other models when replacing feature extraction network backbones. This research can further alleviate challenges in pest and disease identification within smart agriculture, promoting the intelligent transformation of agricultural practices.

## Data Availability

The original contributions presented in the study are included in the article/supplementary material. Further inquiries can be directed to the corresponding author.
